# TCF3 and ID3 Regulate TSPAN32 Expression in Burkitt Lymphoma

**DOI:** 10.1111/sji.70040

**Published:** 2025-06-26

**Authors:** Grazia Scuderi, Katia Mangano, Gian Marco Leone, Paolo Fagone, Ferdinando Nicoletti

**Affiliations:** ^1^ Department of Biomedical and Biotechnological Sciences University of Catania Catania Italy

**Keywords:** Burkitt lymphoma, tetraspanins, TSPAN32

## Abstract

Burkitt lymphoma (BL) is an aggressive non‐Hodgkin B‐cell lymphoma characterised by chromosomal translocations involving the MYC gene, leading to its overexpression and driving uncontrolled proliferation. BL is categorised into endemic, sporadic, and immunodeficiency‐associated subtypes, each with distinct clinical and epidemiological features. TSPAN32, a member of the tetraspanin family, plays a key role in B cell development and immune regulation. In this study, we investigated the regulation of TSPAN32 expression in BL subtypes. Our results show that TSPAN32 expression is significantly downregulated in endemic, sporadic, and HIV‐associated BL. Notably, this downregulation is independent of Epstein–Barr virus (EBV) infection, as no significant differences in TSPAN32 expression were observed between EBV‐positive and EBV‐negative BL clones. Functional studies revealed that overexpression of a wild‐type ID3 gene, a known repressor of TCF3, and knockdown of TCF3, both led to a significant upregulation of TSPAN32, particularly in BL41 and Daudi cells, which harbour ID3 mutations. Supporting this, ChIP‐seq analysis identified TCF3 binding peaks on the TSPAN32 gene, providing mechanistic evidence of its regulation by TCF3. These findings shed light on the complex transcriptional network regulating TSPAN32 and its dysregulation in BL. Overall, our study suggests that TSPAN32 may serve as both a biomarker and a potential therapeutic target for this disease.

## Graphical abstract

TSPAN32 expression is significantly downregulated in endemic, sporadic, and HIV‐associated Burkitt Lymphoma, and it is regulated by the TF3/ID3 pathway.

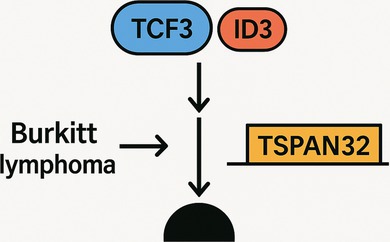

## 1 Introduction

Burkitt lymphoma (BL) is an aggressive mature B‐cell neoplasm. Characterized by uniform, medium‐sized cells with basophilic cytoplasm, multiple small nucleoli, a germinal center B‐cell immunophenotype, a high proliferation rate, and MYC rearrangement involving an immunoglobulin (Ig) gene partner [1]. Additional but not consistently present features of BL include a diffuse growth pattern and a characteristic starry‐sky appearance. Immunophenotypic analysis typically reveals that lymphoma cells express surface light chains, pan B‐cell antigens, CD10, BCL‐6, CD38, and MYC, while lacking expression of BCL‐2 and LMO2. Immunohistochemical evaluation for Ki‐67 demonstrates an almost 100% proliferation index [2, 3].

Based on epidemiologic features, BL is typically divided into three main clinical variants: Endemic form, Sporadic form, and Immunodeficiency‐associated form [1, 4]. The Endemic form is prevalent in Africa and strongly associated with Epstein–Barr virus (EBV) infection, particularly in children. This form often presents with extranodal tumours, typically affecting the jaw, facial bones, kidneys, and ovaries [4]. The Sporadic form is found in Western countries, with a lower association with EBV infection. It primarily affects the intestines, kidneys, and central nervous system [4]. The Immunodeficiency‐associated form is often seen in patients with HIV/AIDS or in immunocompromised individuals. It is similar to the Sporadic form but can be more widespread and severe [4]. Most tumour cells of BL exhibit specific chromosomal translocations involving always chromosome 8 (8q24) and one of chromosomes 14, 2, or 22 (respectively 14q32, 2p12, and 22q11) [5]. The molecular consequence of the three translocations is deregulated expression of the *MYC* oncogene, which has an essential role in cell cycle control [2]. MYC deregulation is crucial but not sufficient for BL development [6, 7]. Recent molecular studies have identified recurrent genetic abnormalities in BL beyond the MYC rearrangement. The main genes and pathways involved include: BCR/PI3K signalling, cell proliferation, G protein‐coupled receptor/sphingosine‐1‐phosphate (S1P) signalling, epigenetic regulation [8, 9, 10, 11]. In particular, TCF3 mutations are present in 10%–20% of cases, while ID3 mutations are observed in 50% [11, 12]. These mutations, rarely found in other B‐cell lymphomas, enhance TCF3 activity, a key regulator of germinal center B‐cell differentiation, thereby promoting BCR and PI3K signalling [12].

BL symptoms vary by subtype but appear rapidly due to its aggressive nature. Endemic BL often presents with jaw or periorbital swelling and abdominal involvement in retroperitoneal tissues, intestines, ovaries, or kidneys [13]. Rarely (15%), it causes paraplegia and incontinence, with jaw involvement most common in children aged 3–7 years [14]. Sporadic BL frequently affects the abdomen (60%–80%), causing pain, distension, nausea, vomiting, or gastrointestinal bleeding. Around 25% of cases involve the ileocecal region, presenting as a mass or pain from intussusception [15]. Other affected areas include the head and neck (e.g., tonsils, sinuses), while jaw involvement is rare [16, 17]. Uncommon sites for all forms include the mediastinum, CNS, skin, testes, breasts, and thyroid.

Treatment protocols such as CODOX‐M/IVAC have demonstrated good clinical responses, achieving 2‐year event‐free survival (EFS) rates of up to 92% in younger individuals [18]. In contrast, older patients often face greater toxicity and reduced survival outcomes [19]. Adaptations of paediatric regimens, including Hyper‐CVAD and R‐EPOCH, have been tailored for adults, delivering better results with lower toxicity, especially in those with low‐risk disease [20]. The inclusion of rituximab in treatment regimens has significantly enhanced outcomes [21, 22].

Tetraspanin 32 (TSPAN32) is a unique member of the tetraspanin superfamily, primarily expressed in haematopoietic tissues. It plays a critical role in immune regulation and is implicated in several autoimmune diseases. It is structurally unique, lacking typical features like the A/GFLGC motif and possessing an extended cytoplasmic domain. TSPAN32 plays a role in controlling immune responses and is implicated in several autoimmune diseases, including multiple sclerosis (MS) [21, 22], Rheumatoid Arthritis (RA) [23] and systemic lupus erythematosus (SLE) [24]. In MS, TSPAN32 regulates T cell activity, with decreased expression linked to disease progression and immune dysregulation. In SLE, type I interferon pathways reduce TSPAN32 expression, correlating with increased disease severity. Genome‐wide studies have also associated TSPAN32 variants with other autoimmune conditions such as inflammatory bowel disease (IBD) and systemic sclerosis (SSc), emphasising its role in immune system regulation [25].

The present study aims to investigate the regulation of TSPAN32 expression in BL. By examining the transcriptional mechanisms underlying TSPAN32 dysregulation across endemic, sporadic, and HIV‐associated BL subtypes, we seek to identify its role in lymphoma pathogenesis. The study also explores the involvement of key regulatory factors, such as MYC, TCF3, and ID3, as well as the potential role of Epstein–Barr virus (EBV) in modulating TSPAN32 expression.

## 2 Materials and Methods

### 2.1 TSPAN32 Expression Analysis in B Cells and in BL

Analysis of TSPAN32 expression in the different stages of maturation of murine B cells was performed by interrogating the GSE15907 microarray dataset [26]. GSE15907 was generated as part of the Immunological Genome Project (ImmGen). Briefly, primary cells from multiple immune lineages were isolated ex vivo from young adult C57/B6 male mice (*n* = 3) and double‐sorted to yield > 99% purity. RNA was extracted and whole‐genome transcriptomic levels were obtained using the Affymetrix 1.0 ST MuGene array platform (Santa Clara, California, US). Pre‐preprocessing and normalisation of the raw data was performed using the standard RMA workflow (background adjustment, quantile normalisation, median polish probeset summarization). The cell populations included: Common Lymphoid Progenitor (Lin‐AA4+Kit+IL7Ra+B220‐) from Bone marrow; Pre‐pro‐B (Lin‐AA4+Kit+IL7Ra+B220+) from Bone marrow; Pro‐B (AA4+IgM‐CD19+CD43+HSA+) from Bone marrow; Cycling pre‐B (AA4+IgM‐CD19+CD43+HSA++) from Bone marrow; Pre‐B (AA4+IgM‐CD19+CD43‐HSA+) from Bone marrow; Newly‐formed B (AA4+IgM+CD19+HSA+) from Bone marrow; T1 (transitional) (CD19+B220+IgM++AA4+CD23‐) from Spleen; T2 (transitional) (CD19+B220+IgM++AA4+CD23+) from Spleen; T3 (transitional) (CD19+B220+IgM+AA4+CD23+) from Spleen; Follicular (CD19+B220+IgM+AA4‐CD23+) from Spleen; Germinal Center B cells (CD19+IgM+IgD‐GL7+PNA+) from Spleen; Marginal Zone (CD19+B220+IgM+AA4‐CD23‐CD21/35+) from Spleen; B‐1a (CD19+B220+IgM+AA4‐CD23‐CD43+) from Spleen; Recirc B (AA4‐IgM+CD19+) from Bone marrow.

In order to evaluate the expression levels of TSPAN32 in BL, in comparison to normal B cells, we interrogated the GEO database and the dataset GSE43677 was selected as it contained transcriptomic profiles of naive B cells (*n* = 8), germinal center (GC) B cells (*n* = 13), eight endemic BL cell lines (Daudi, Namalwa, Raji, CA‐46, DG‐75, Namalwa, Raji, Ramos), an HIV‐associated BL (DoGKit), and four sporadic BL cell lines (DoGum, Gumbus, BL‐41 and BLUE‐1). The dataset was generated using the Affymetrix Human Genome U133A Array. Raw data were normalised by the variance stabilisation method [27].

### 2.2 Regulation of TSPAN32 Expression in BLs

In order to investigate the role of EBV infection on the expression levels of TSPAN32, we analysed the GSE100458 dataset [28]. The dataset included whole‐genome gene expression profiles of EBV‐positive and EBV‐loss clones derived from four endemic BL cell lines. The Affymetrix Human Genome U133 Plus 2.0 Array was used for the generation of the dataset.

BL (BL) is an aggressive B‐cell non‐Hodgkin lymphoma characterised by the deregulation of several key oncogenes and tumour suppressors. The MYC oncogene is the hallmark driver of BL, where chromosomal translocations place MYC under the control of immunoglobulin gene enhancers, leading to its constitutive overexpression. Two other critical drivers in BL are TCF3 (E2A) and ID3, which play pivotal roles in B‐cell development and differentiation. TCF3 is a transcription factor essential for the maintenance of the germinal center B‐cell phenotype. It regulates the expression of numerous genes involved in B‐cell survival and proliferation. Activating mutations in TCF3, seen in a subset of BL cases, enhance its transcriptional activity, promoting cell growth. ID3, an inhibitor of TCF3, acts as a negative regulator by sequestering TCF3 in an inactive form. In BL, ID3 is often mutated, with both missense and truncating mutations disrupting its function. These ID3 mutations lead to unchecked TCF3 activity, which cooperates with MYC overexpression to drive lymphomagenesis.

In order to determine whether MYC is involved in TSPAN32 regulation in BL, we selected the datasets GSE199925 and GSE135800 [29]. The GSE199925 dataset included whole‐genome expression data of ST486 BL cells infected with two different anti‐Myc shRNAs or a non‐targeting control shRNA (Day 8 post‐infection). The GSE135800 dataset includes the transcriptome profiling of BLlymphoblastoid P493‐6 cells treated with MYCi975, and P493‐6 treated with tetracycline to turn off MYC expression.

### 2.3 ID3 Overexpression and TCF3 shRNA

Namalwa, Daudi, and BL‐41 BL cell lines were cultured in RPMI 1640 medium supplemented with 10% fetal bovine serum (FBS) and 1% penicillin–streptomycin and maintained in a humidified incubator at 37°C with 5% CO_2_. ID3 expression vectors were generated by amplifying ID3 cDNAs via PCR using the primers 5′‐CTGGATCCGCCACCATGAAGGCGCTGAGCCCGGTGC‐3′ (forward) and 5′‐GACTCGAGTTATCAGTGGCAAAAGCTCCTTTTGTCG‐3′ (reverse) and cloning the amplified product into the pCMV or pMSCV retroviral backbone using BamHI and XhoI restriction sites. For TCF3 knockdown, BL cell lines were transduced with the pLKO.1‐shRNA vector carrying the sequence GAGCGGAACCTGAATCCCAAA or with a scrambled control vector. To study the effect of ID3 overexpression on TSPAN32 expression, cells were transduced with either a pMSCV‐ID3‐WT expression vector or an empty control vector. Transduced cells were selected using puromycin at a concentration of 2 μg/mL for 3–7 days to ensure stable integration. Gene expression or shRNA knockdown was induced by adding doxycycline at a final concentration of 50 ng/mL to the culture medium for the required experimental time points.

Cell proliferation under vector induction in the three BL cell lines (Namalwa, Daudi, and BL41) was evaluated using the MTT assay. Cells were seeded in 96‐well plates at 2 × 10^5/well. After an incubation (24–72 h) period, MTT reagent was added, and cells were incubated to allow for formazan crystal formation. Absorbance was measured at 570 nm using a microplate reader to assess cellular proliferation. Experiments were performed in technical triplicate to ensure reproducibility. Data are presented as a percentage of optical density (OD) relative to control cells, which were set at 100% to normalise the results.

### 2.4 ChiP‐Seq Analysis

TCF3 binding to the TSPAN32 gene was investigated using ChIP‐Seq analysis of the publicly available SRA052618 dataset. Briefly, 36 bp sequence tags were aligned to the human genome (hg18) using the ELAND_EXTENDED software. After removing redundant reads, uniquely mapping reads with a maximum of two mismatches were used for further analysis. To identify binding sites (“peak calling”), the genome was divided into 25 bp bins. Each tag was extended by 200 bp along the direction of its read to match the average library size. Tags with fewer than three hits in a bin were discarded. Consecutive significant bins were merged to form “peaks”, with the apex being the bin with the most hits. Control experiments using empty vectors were used to exclude artefact peaks. Peaks were associated with genes if their apex was located within the gene body or within 10 kb upstream.

### 2.5 Statistical Analysis

Data are presented as Mean ± SD and statistical analysis was performed using either a Student's *t*‐test or one‐way ANOVA followed by Fisher's LSD multiple test comparison. The GraphPad Prism software was used for the statistical analysis and generation of the graphs. The GSE43677 dataset was used for the identification of the genes both positively and negatively correlated to TSPAN32 in Germinal Center B cells and BL cells lines. The Pearson's correlation r was calculated and the genes with *r* ≥ |0.5| and FDR ≤ 0.05 were chosen for the identification of the biological pathways commonly regulated with TSPAN32, using the web‐based utility, Metascape [30]. All experimental assays, including MTT‐based proliferation studies, were performed in technical triplicates to ensure reproducibility. Results were normalised relative to control groups and expressed as percentages, with statistical significance determined as described above.

## 3 Results

### 3.1 TSPAN32 Expression in Murine B Cell Maturation

**FIGURE 1:**
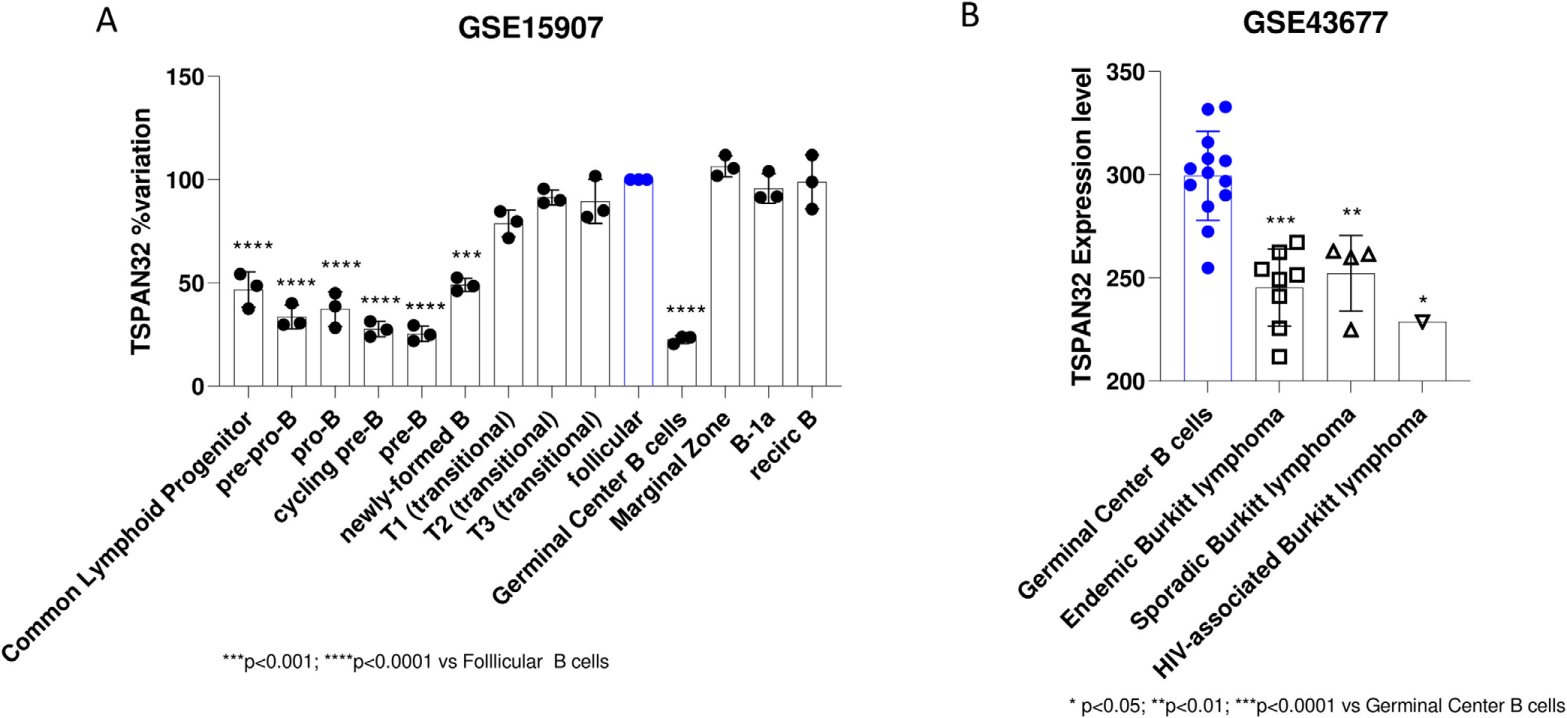
TSPAN32 expression during the maturation of murine B cells. Gene expression analysis of TSPAN32 was performed by interrogating the GSE15907 microarray dataset (A). Expression levels of TSPAN32 in BL cell lines, as compared to normal Germinal Center B cells, as determined by interrogating the GSE43677 dataset (B).

To examine the expression of TSPAN32 during various stages of murine B cell maturation, we compared its levels in follicular B cells against multiple B cell progenitor and mature stages. The results revealed significant differences in TSPAN32 expression across various stages. Follicular B cells displayed a higher TSPAN32 expression compared to early progenitor stages, such as Common Lymphoid Progenitors (CLP) (*p* < 0.0001), and pre‐pro‐B cells (*p* < 0.0001). Similarly, significant differences were observed when comparing follicular B cells to pro‐B cells (*p* < 0.0001), cycling pre‐B cells (*p* < 0.0001), and pre‐B cells (*p* < 0.0001) (Figure 1A).

Interestingly, TSPAN32 expression in follicular B cells was also significantly higher when compared to more differentiated stages, including newly‐formed B cells (*p* < 0.0001), T1 transitional B cells (*p* = 0.0005), and germinal center B cells (*p* < 0.0001). However, no significant differences were observed between follicular B cells and T2 transitional B cells, T3 transitional B cells, marginal zone B cells, B‐1a B cells, and recirculating B cells.

### 3.2 TSPAN32 Expression in BL

To investigate the differential expression of TSPAN32 mRNA between BL and Germinal Center B cells, we performed a comparative analysis across various BL subtypes (Figure 1B). The comparison between Germinal Center B cells and endemic BL revealed a highly significant reduction in TSPAN32 expression in endemic BL. Similarly, in sporadic BL, we observed a significant downregulation of TSPAN32. Lastly, the comparison between Germinal Center B cells and HIV‐associated BL showed a similar trend of reduction (Figure 1B).

### 3.3 Pathway and Transcription Factor Enrichment Analysis

**FIGURE 2:**
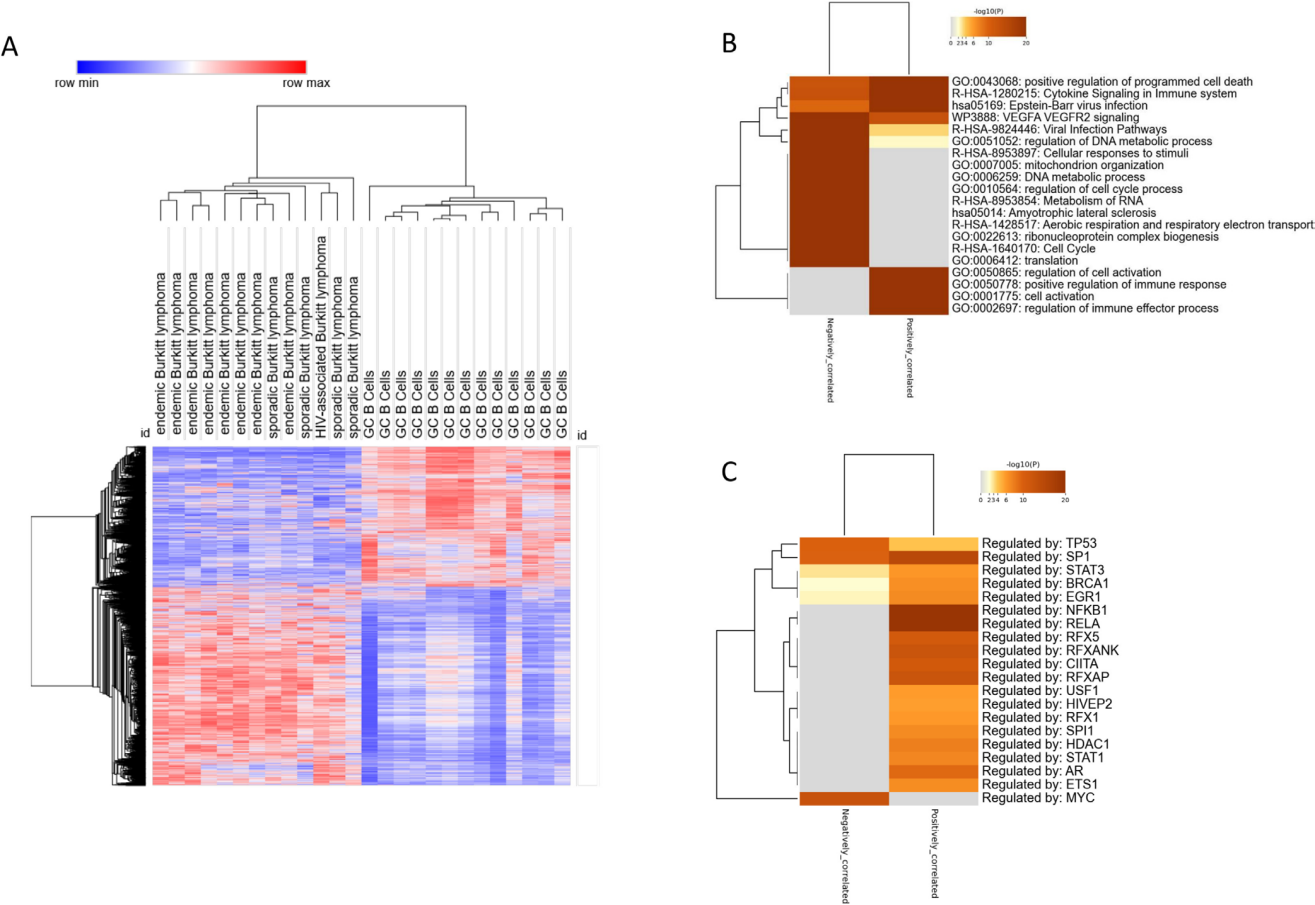
Heatmap of the genes positively and negatively correlating with TSPAN32 transcriptional levels, as determined by interrogating the GSE43677 dataset (A). Heatmap of the most significant biological processes enriched by the genes positively and negatively correlating with TSPAN32, as determined using the Metascape software (B). Heatmap of the Transcription Factors putatively involved in the regulation of the genes positively and negatively correlating with TSPAN32, as determined using the Metascape software (C).

By interrogating the GSE43677 dataset, we identified the genes positively and negatively correlated with TSPAN32 expression in Germinal Center B cells and BL cell lines. We identified 2059 positively correlated and 2990 negatively correlated genes (Figure 2A). Pathway enrichment analysis of these genes revealed significant involvement in multiple biological processes and signalling pathways (Figure 2B). Functional enrichment analysis revealed a highly significant overrepresentation of pathways and biological processes related to RNA metabolism, immune signalling, and cell cycle regulation. Among the top enriched terms, “Metabolism of RNA” (Reactome: R‐HSA‐8953854) and “ribonucleoprotein complex biogenesis” (GO:0022613) showed the highest significance with ‐log(q‐values) of 95.657 and 94.373, respectively. Immune‐related pathways, such as “Cytokine Signaling in Immune System” (Reactome: R‐HSA‐1280215), “positive regulation of immune response” (GO:0050778), and “Epstein‐Barr virus infection” (KEGG: hsa05169), were also prominently enriched, indicating a strong immunological component. Additionally, pathways related to DNA metabolism, cell activation, and programmed cell death, such as “DNA metabolic process” (GO:0006259) and “positive regulation of programmed cell death” (GO:0043068), were significantly overrepresented.

Next, we examined the transcription factors that regulate the genes positively and negatively correlating with TSPAN32 expression in BL. Transcription factor enrichment analysis identified MYC (logP = −13.0) as the most significantly enriched among negatively correlated genes. Other notable factors with strong enrichment in this group included TP53 (logP = −9.9) and SP1 (logP = −9.8). For positively correlated genes, the strongest enrichment was observed for SP1 (logP = −15.0), followed by STAT3 (logP = −6.2) and MYCN (logP = −4.7). A subset of transcription factors, including TP53, SP1, E2F1, MYCN, ESR1, YY1, STAT3, HDAC2, BCL6, and JUN, showed enrichment in both negatively and positively correlated gene sets(Figure 2C).

### 3.4 TSPAN32 Transcriptional Regulation

**FIGURE 3:**
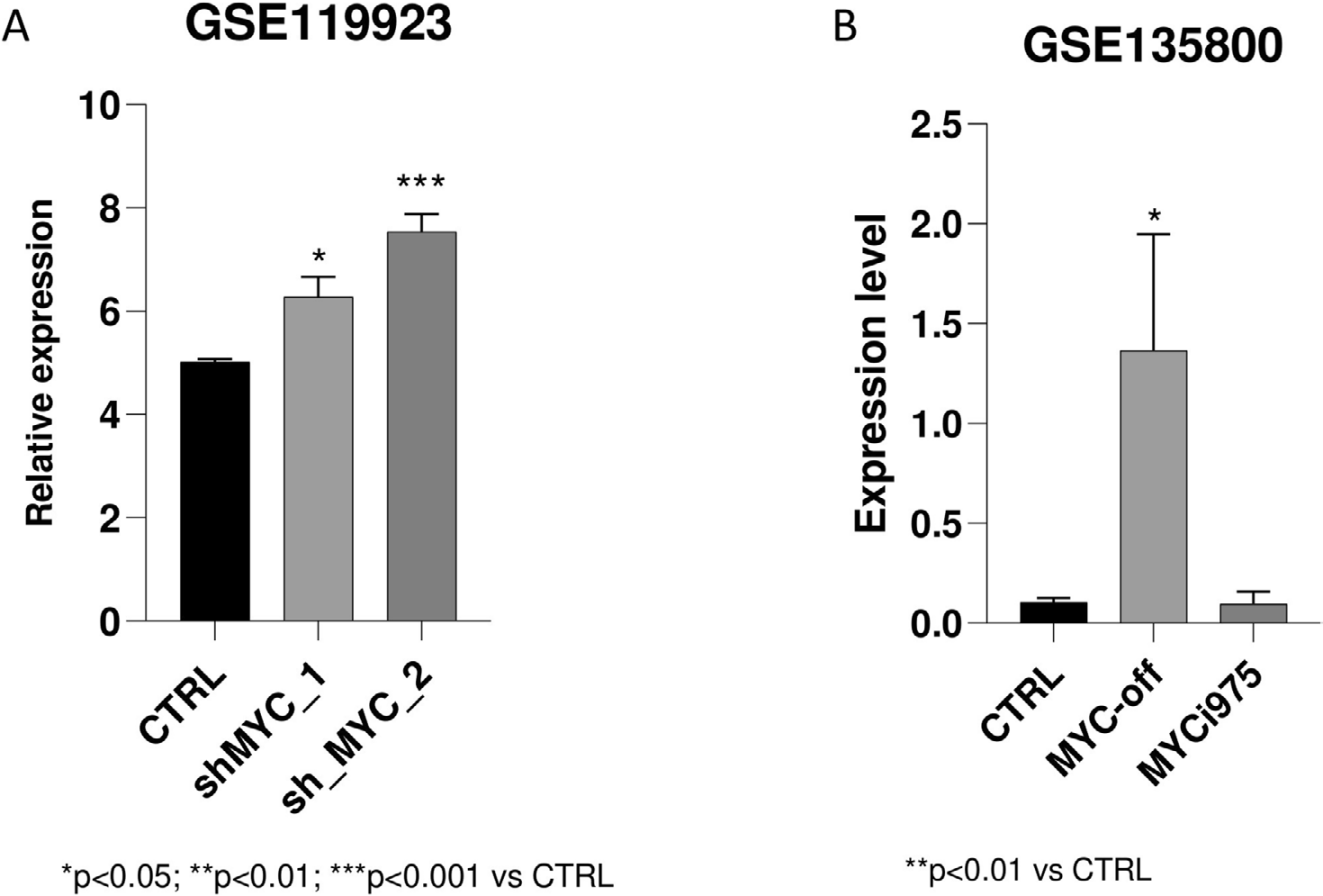
Effect of MYC modulation on TSPAN32 transcriptional levels. Effect of two different shMYC constructs on TSPAN32 mRNA levels, as determined in the GSE119925 dataset (A). Effect of MYC silencing (MYC‐off) and of a MYC inhibitor (MYCi975) on TSPAN32 mRNA levels, as determined using the GSE135800 dataset (B).

BLBLBased on transcription factor prediction results that identified MYC as a key regulator, we investigated its impact on TSPAN32 expression by analysing the GSE119923 and GSE135800 datasets. We observed that silencing MYC significantly increased TSPAN32 expression. In particular, we observed in the GSE119923 dataset a significant increase in TSPAN32 levels between the control cells and the cells treated with two different shMYC constructs (adjusted *p* value = 0.0480, and adjusted *p* value = 0.0070, respectively) (Figure 3A). Furthermore, by interrogating the GSE135800 dataset, we observed that silencing MYC was associated with a significant upregulation of TSPAN32 expression (adjusted *p* value = 0.0092) (Figure 3B). Unexpectedly, however, no significant difference was found between the control cells and the cells treated with the MYC inhibitor, MYCi95 (Figure 3B). Despite these results suggesting a role for MYC in regulating TSPAN32 expression, however, previously published ChIP‐Seq data indicate that the TSPAN32 gene is not a direct target of MYC [31]. Therefore, in order to better investigate the regulatory factors controlling TSPAN32, we next focused on two additional known driver genes in BL: TCF3 and ID3.

**FIGURE 4:**
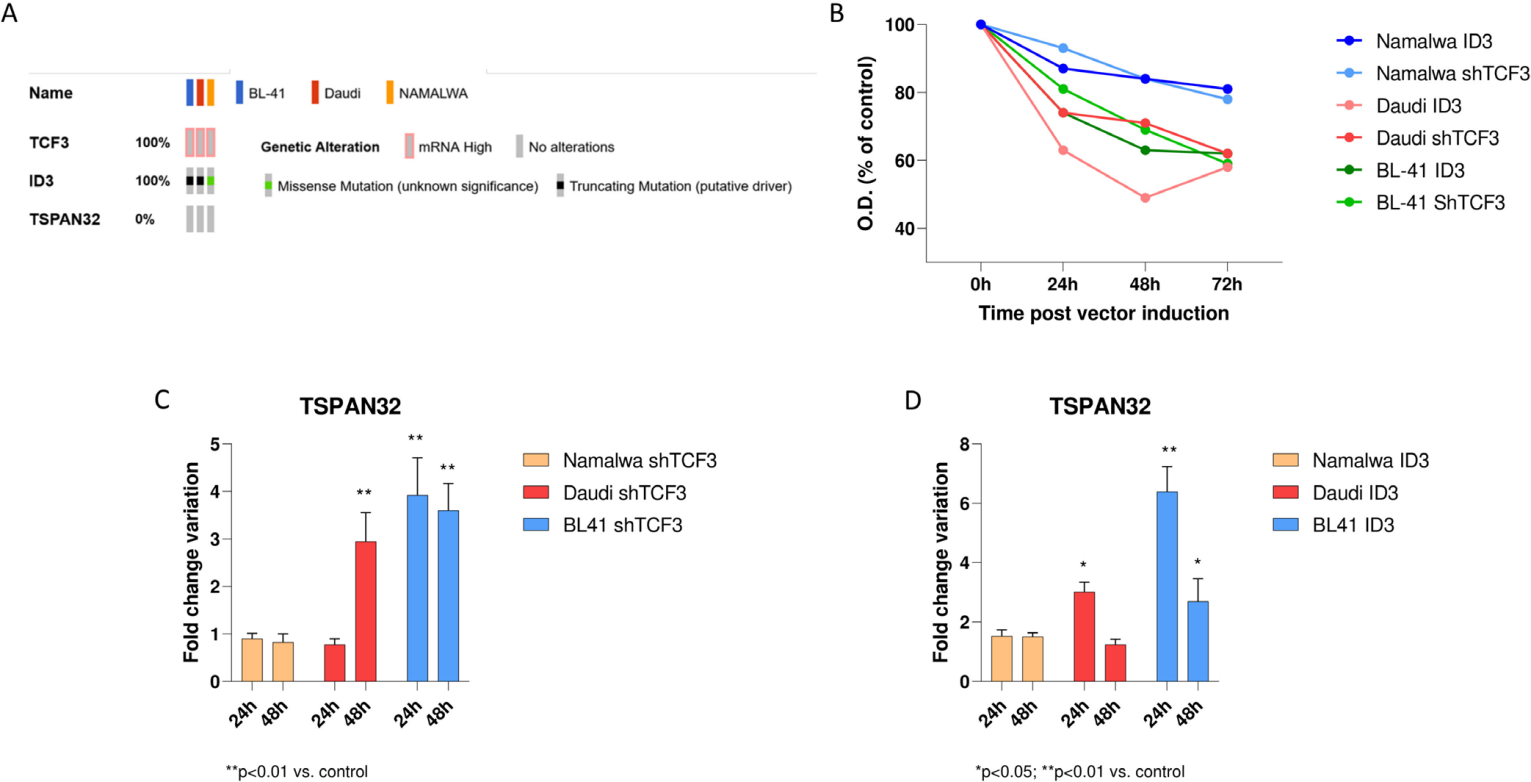
Effects of TCF3 and ID3 in Burkitt cell lines. Genomic characteristics of the BL cell lines BL‐41, Daudi and Namalwa (A). Proliferation of BL‐41, Daudi and Namalwa cells upon TCF3 and ID3 modulation (B). Expression levels of TSPAN32 mRNA levels in BL‐41, Daudi and Namalwa upon shTCF3 treatment (C). Expression levels of TSPAN32 mRNA levels in BL‐41, Daudi and Namalwa upon ID3 overexpression (D).

To this aim, we investigated the modulation of TSPAN32 from three BL cell lines (Namalwa, Daudi, and BL41) under the modulation of TCF3 and ID3. All three cell lines exhibit high expression levels of TCF3, and Daudi and BL‐41 cell lines harbour truncating mutations in ID3 (Figure 4A; data retrieved from cBioportal). Knockdown of TCF3 and induction of a functional ID3 gene expression were associated with a reduced proliferative ability of all three cell lines, with the most pronounced effect for the Daudi and BL‐41 cells (Figure 4B). PCR analysis confirmed the efficacy of the genetic manipulations, showing a mean decrease of 64.6% in TCF3 expression and a 31.1% increase in ID3 expression (data not shown). In the Namalwa cell line, knockdown of TCF3 using shRNA (at 24 and 48 h) resulted in a not significant modulation of TSPAN32. Also, overexpression of a functional ID3 gene at 24 and 48 h led to a modest, although not significant, upregulation of TSPAN32 (Figure 4C,D).

In the Daudi cell line, shTCF3 treatment revealed a modest downregulation of TSPAN32 at 24 h, followed by a significant upregulation at 48 h. Upon ID3 modulation in Daudi, a significant upregulation of TSPAN32 was observed at 24 h, which remained elevated at 48 h (Figure 4C,D).

In the BL41 cell line, the knockdown of TCF3 resulted in the highest observed upregulation of TSPAN32 among the three cell lines, at both 24 and 48 h. Notably, modulation of ID3 in BL41 resulted in an even more significant upregulation of TSPAN32 at 24 h (Figure 4C,D).

Analysis of ChIP‐seq data from the SRA052618 dataset confirmed our in vitro data regarding the transcriptional regulation of TSPAN32 in BL cell lines (Namalwa and BL41) (Table 1). Several ChIP‐seq peaks for TCF3 and ID3 located near or within the TSPAN32 gene were identified in BL41 and Namalwa cell lines. Peak 20779, located approximately 2807 bp upstream of the transcription start site (TSS), suggests it could be part of a promoter or enhancer region directly involved in regulating TSPAN32 transcription. The ChIP‐seq tag maxima for this peak are similar in both BL41 and Namalwa, suggesting comparable chromatin interactions in both lines. Its proximity to the TSS makes it a strong candidate for contributing to observed changes in TSPAN32 expression following TCF3 and ID3 modulation, while its moderate ChIP‐seq tag density points to involvement in basal regulation. Peak 20783, located 12,407 bp downstream of the TSS, could represent a distal regulatory element such as an enhancer. Peak 20784, located 14,457 bp downstream of the TSS, also likely represents a distal enhancer, with significantly higher ChIP‐seq tag density in Namalwa [32] compared to BL41 [15] Table 1.

**TABLE 1:** TCF3 ChIP‐Seq Peak Calling for the TSPAN32 gene in BL41 and Namalwa cells, as determined from the SRA052618 dataset.

Chromosome	Peak start	Peak end	ChlPseq Tag Max (BL41)	ChlPseq Tag Max (Namalwa)	Gene Symbol	Distance to TSS
11	2282625	2282649	14	13	TSPAN32	2807
11	2292100	2292374	21	22	TSPAN32	12407
11	2294275	2294299	15	38	TSPAN32	14457

## 4 Discussion

Our findings suggest that TSPAN32 expression is carefully regulated during B cell maturation and is significantly altered in BLBL, pointing to its potential role in both normal immune function and malignancy. The present work complements previous observations by our group and others, regarding the role of TSPAN32 in immune cell regulation. TSPAN32, a member of the tetraspanin family [33, 34, 35], has been implicated in diverse biological processes, particularly those involving immune cell differentiation, activation, and function. Earlier studies have demonstrated that TSPAN32 plays a critical role in maintaining immune homeostasis, and its dysregulation has been associated with altered immune responses and susceptibility to autoimmune diseases and haematopoietic cancer [21, 22, 23, 32, 35, 36].

One of the most striking observations in our study is the dynamic expression of TSPAN32 across different B cell maturation stages. Its significant upregulation in follicular B cells, compared to earlier progenitor stages (CLP, pre‐pro‐B, and pro‐B cells), suggests an important function in transitioning to more mature B cell states. Interestingly, while TSPAN32 expression remains high in follicular B cells, it declines significantly in germinal center B cells. Given that the Germinal Center is a hub of intense proliferation, somatic hypermutation, and class‐switch recombination, this downregulation could indicate that TSPAN32 is not required—or may even be actively repressed—during these processes. It is worth exploring whether the loss of TSPAN32 at this stage enables the flexibility needed for affinity maturation, or whether its absence helps facilitate the characteristic gene expression changes that define germinal center B cells.

The comparison between BLs and Germinal Center B cells reveals a consistent and significant downregulation of TSPAN32 in endemic, sporadic, and HIV‐associated BL. This finding supports the notion that TSPAN32 dysregulation is a common feature across various forms of BLBL. The highly significant reduction in TSPAN32 expression across all subtypes indicates that TSPAN32 loss may contribute to the malignant phenotype. Given the role of TSPAN32 in B cell maturation, its downregulation in BLBL suggests a potential loss of its regulatory functions, which could be involved in controlling cell proliferation, apoptosis, or cell cycle regulation.

The reduced expression of TSPAN32 across these subtypes is in line with the aggressive nature of BL, which is characterised by rapid proliferation and a high mitotic index. TSPAN32 may act as a tumour suppressor, with its downregulation contributing to uncontrolled proliferation. Alternatively, TSPAN32 might be involved in the regulation of key signalling pathways or cellular processes, whose dysregulation promotes the oncogenic phenotype observed in BL.

The pathway enrichment analysis of genes correlating with TSPAN32 provides further insights into the biological processes that may be co‐regulated with TSPAN32 expression in both normal and malignant B cells. The enrichment of pathways involved in cell cycle regulation suggests that TSPAN32 may influence cell proliferation and genomic stability. Dysregulation of these processes is a hallmark of cancer, and the involvement of TSPAN32 in these pathways underscores its potential role in suppressing malignant transformation.

The identification of transcription factors, including MYC, TP53, and SP1, associated with genes negatively correlating with TSPAN32 provides additional data about the regulatory mechanisms governing TSPAN32 expression in BL. MYC, a pivotal oncogene frequently dysregulated in this malignancy, is instrumental in promoting cell proliferation and metabolic reprogramming. MYC silencing significantly upregulates TSPAN32 expression, suggesting a regulatory effect. Despite this, the absence of direct MYC binding to the TSPAN32 promoter, as demonstrated by prior ChIP‐seq studies, implies that MYC modulates TSPAN32 expression through intermediary factors or epigenetic modifications.

Interestingly, MYCi975, a MYC inhibitor that disrupts the interaction of MYC with its partner MAX, does not alter TSPAN32 expression. This suggests that the effect of MYC on TSPAN32 is not mediated through the MYC/MAX complex but it could be rather dependent on the interaction of MYC with alternative partners, such as MIZ1. MYC and MIZ1 together act as a repressive transcriptional complex, a mechanism that might explain the influence of MYC on TSPAN32 [29, 37, 38].

It is important to mention that a previous study [39] demonstrated that inhibition of MYC in BL cell lines is associated with an upregulation of both IFN‐β and IFN‐α2. These findings are in contrast to our earlier data on B cells from patients with systemic lupus erythematosus (SLE) [24], where type I interferons lead to a downregulation of TSPAN32. This apparent discrepancy is likely dependent to differences in the regulatory networks between malignant B cells and normal activated B cells in autoimmune conditions. Indeed, in BL, the regulation of TSPAN32 seems more complex and may involve additional intermediary factors influenced by MYC. The unique genetic and transcriptional landscape of BL complicates the relationship between MYC, type I interferons, and TSPAN32, which warrants the need for additional studies to delineate these context‐specific regulatory pathways.

Finally, our study investigated the relationship between TSPAN32 expression and the BL‐associated transcription factors TCF3 and ID3. Both of these factors are known to play key roles in the pathogenesis of BL. The upregulation of TSPAN32 following ID3 modulation, particularly in BL41 and Daudi cell lines, suggests that ID3 mutations lead to a loss of repressive function, thereby allowing for the upregulation of TSPAN32. This is particularly evident in the BL41 cell line, where truncating mutations in ID3 result in a pronounced upregulation of TSPAN32, supporting the idea that wild‐type ID3 acts as a repressor of TCF3 and its downstream targets, including TSPAN32. Supporting our in vitro data, the identification of ChIP‐seq peaks for TCF3 near the TSPAN32 gene further strongly suggest its direct role in modulating the expression of TSPAN32 in BL. The proximity of peak 20779 to the TSS suggests it could represent a promoter or enhancer region that directly influences TSPAN32 transcription and the moderate ChIP‐seq tag maxima at this peak indicates that it may contribute to the basal regulation of TSPAN32. Also, the distal peaks (20783 and 20784), may allow the interaction with the TSPAN32 promoter through chromatin looping, facilitating the recruitment of other transcriptional activators or repressors.

While our study provides novel data on the potential role of TSPAN32 in BL, there are notable limitations. A key limitation is the lack of direct functional validation of the role of TSPAN32 in BL cells from real‐world patients. While our findings suggest that TSPAN32 dysregulation is associated with the malignant phenotype, definitive proof of its direct regulatory impact would require further functional assays. Also, CRISPR‐mediated disruption of TCF3 binding sites would be necessary to fully elucidate its contribution to the disease process.

## 5 Conclusion

In conclusion, our study demonstrates that TSPAN32 expression is tightly regulated during B cell maturation and is significantly dysregulated in BL. The downregulation of TSPAN32 across BL subtypes suggests its potential role as a tumour suppressor gene. The involvement of TSPAN32 in pathways related to cell cycle control, DNA repair, RNA metabolism, and mitochondrial function supports its possible role in regulating the proliferative and metabolic state of B cells. The identification of key transcription factors, such as MYC, TCF3, and ID3, sheds light on the regulatory networks that control TSPAN32 expression in BL. Future studies should focus on elucidating the precise molecular mechanisms through which TSPAN32 contributes to B cell development and BL pathogenesis, as well as the therapeutic potential of targeting TSPAN32 dysregulation in these types of lymphomas.

## Author Contributions

Grazia Scuderi, Paolo Fagone, Ferdinando Nicoletti conceived and designed the study; Grazia Scuderi, Gian Marco Leone performed experiments and collected the data; Grazia Scuderi, Paolo Fagone analysed the data; Katia Mangano, Paolo Fagone, Ferdinando Nicoletti interpreted the data; Grazia Scuderi, Paolo Fagone wrote the paper. All the authors approved the final version of the manuscript.

## Disclosure

The authors have nothing to report.

## Data Availability

The data that support the findings of this study are available from the corresponding author upon reasonable request.
